# Feature-Guided Machine Learning for Studying Passive Blood–Brain Barrier Permeability to Aid Drug Discovery

**DOI:** 10.3390/ijms262211228

**Published:** 2025-11-20

**Authors:** Baining Zhu, Suwei Liu

**Affiliations:** 1 Phillips Exeter Academy, Exeter, NH 03833, USA; bzhu2@exeter.edu; 2 Mechanical Engineering, Northwestern University, Evanston, IL 60208, USA

**Keywords:** blood–brain barrier, machine learning, drug discovery, permeability prediction

## Abstract

Effective prediction of blood–brain barrier (BBB) permeability remains essential for central nervous system drug development. This study evaluates multiple supervised machine learning models using a public dataset of permeable and non-permeable compounds. Random Forest models demonstrate optimal balance between accuracy and generalizability, outperforming more complex gradient boosting methods that were prone to overfitting. Feature analysis identifies NH/OH and NO group counts as key determinants of passive diffusion, with reduced hydrogen bond donor and heteroatom counts enhancing permeability. Additionally, model performance deteriorates at NH/OH count = 3, establishing this as a decision boundary where hydrogen bonding complexity disrupts reliable prediction. This study shows the non-linear structure-permeability relationships that challenge traditional descriptor-based approaches, while demonstrating that machine learning can simultaneously provide both accurate prediction and applicable insights for drug discovery applications.

## 1. Introduction

The blood–brain barrier (BBB) plays a vital role in the central nervous system (CNS) by regulating the exchange of molecules between the bloodstream and neural tissue [1,2,3,4]. However, this protective function has a negative impact on developing therapeutics for neurological disorders such as Alzheimer’s disease, Parkinson’s disease, epilepsy, and brain cancers [5,6,7,8]. Failure to accurately predict BBB permeability has led to significant costs throughout the drug discovery procedures, as promising drug candidates are frequently abandoned late in clinical development. The ability to determine whether a compound can cross the BBB is therefore central to efficient drug discovery [9,10,11,12,13].

A range of experimental strategies has been developed to study BBB transport. In vivo animal models, in vitro cell culture systems, and receptor-mediated assays offer high biological fidelity and provide detailed insights into molecular pathways [14,15,16,17,18]. These approaches produce physiologically relevant data and proof-of-concept validation, but they remain slow, expensive, and difficult to scale up. More specifically, extrapolating results from a controlled lab environment to the complex human BBB is often uncertain, which limits large-scale or early-stage drug discovery [19,20,21].

To increase throughput, theoretical models have been developed to predict permeability using chemical descriptors. More specifically, these descriptors include quantitative structure–activity relationship (QSAR) analysis and features such as molecular weight, lipophilicity, and polar surface area, which are used to estimate transport potential [22,23,24,25,26,27,28,29,30,31]. These methods are computationally efficient and easy to interpret, allowing large compound libraries to be screened at low cost. However, traditional QSAR and linear regression approaches rely on linear additivity assumptions, where molecular features are assumed to contribute independently to permeability without synergistic or antagonistic interactions. This dependence on simplified feature–permeability relationships restricts their ability to capture the complex, non-linear interactions that determine BBB transport, such as threshold effects or compensatory mechanisms between lipophilicity and hydrogen bonding capacity [19,32,33,34].

At the molecular scale, simulation methods such as molecular dynamics (MD) have enabled atomistic views of drug–membrane interactions. MD can describe conformational changes, energetics, and transient membrane phenomena that are difficult to observe experimentally [35,36,37,38]. These studies offer a valuable understanding at the molecular level, but the heavy computational cost makes MD studies suitable for only a small number of molecules. For large compound libraries, MD is not yet a practical tool for permeability prediction [35,39]. Typical MD investigations are limited to small or moderately sized molecules (usually below 400 Da) with well-parameterized chemical groups, since accurate force-field parameterization and extensive sampling times (hundreds of nanoseconds) are required within explicit lipid bilayers [40,41]. Scaling such atomistic systems to thousands of compounds remains computationally prohibitive, as discussed in previous BBB simulation studies [42,43].

To solve the aforementioned bottlenecks, the machine learning (ML) method bridges the gap, combining both the scalability and the ability to learn nonlinear patterns in molecular features [44,45,46]. By training on experimental data, ML models can identify structural and physicochemical properties that correlate with BBB penetration and apply those rules to new compounds [47,48]. In this study, the publicly available Blood–Brain Barrier Penetration (BBBP) dataset from MoleculeNet [49] is used to evaluate the performance of different ML approaches. The dataset contains 1955 compounds annotated as permeable (BBB+) or non-permeable (BBB−), represented by 2048-bit Morgan fingerprints and 208 RDKit descriptors. The data are imbalanced, with 76% labeled BBB+ and 24% BBB−. The BBBP dataset includes a diverse collection of marketed and experimental molecules, including both CNS-targeted drugs and non-CNS agents with reported central side effects. Because permeability labels were derived from experimentally measured log BB values rather than pharmacological indication, the dataset reflects general physicochemical diversity rather than a therapeutic-class bias.

The goal of this study is to provide a comprehensive comparison of machine learning algorithms for predicting BBB permeability, with a focus on further interpretation of the ML performance results. A variety of supervised ML algorithms are examined, ranging from simple linear classifiers such as logistic regression to ensemble-based approaches including random forest and gradient boosting, as well as neural-network-based models. Multiple resampling methods, including Synthetic Minority Oversampling Technique (SMOTE) [50], Borderline SMOTE [51], and combined undersampling, are applied to address the imbalanced feature of the obtained dataset. This work demonstrates how accessible machine learning strategies can inform the rational design of CNS-active compounds and provide an efficient complement to experimental, theoretical and simulation-based approaches.

## 2. Results and Discussions

### 2.1. Base Model Comparison

The dataset used in this study comprises 1955 chemical compounds from the MoleculeNet BBBP dataset. After preprocessing to remove low-variance features (variance threshold of 0.14) and those with missing or infinite values, the dimensionality of the feature space is reduced to 743, which suggests these are the most informative molecular descriptors.

A major characteristic of the dataset is its class imbalance, where 1492 compounds (76.3%) are labeled as BBB+ while 463 (23.7%) are labeled as BBB−. This imbalance ratio of approximately 3:1 introduces bias risks, as classifiers may favor the majority class and inflate accuracy at the expense of minority class prediction. In drug discovery applications, such misclassification is problematic, particularly when false negatives lead to premature exclusion of promising candidates.

To establish baseline predictive performance, 11 machine learning models are trained and evaluated using stratified ten-fold cross-validation. Performance metrics include accuracy, precision, recall, F1-score, and runtime, as summarized in Table 1. The models represent diverse families, including linear models, ensemble methods, boosting frameworks, neural networks, decision trees, probabilistic classifiers, and simple baselines.

Across models, Logistic Regression and Random Forest achieve the best overall balance of precision and recall, with F1-scores of 0.925 and 0.924, respectively. Logistic Regression offers the highest precision (0.891), while Random Forest delivers the highest recall (0.978) and the lowest runtime (0.04 s), making both suitable candidates for large-scale screening applications. Gradient boosting frameworks such as XGBoost, Gradient Boosting, and LightGBM also perform competitively but do not exceed the simpler models in this task. Neural networks and boosting methods achieve strong recall but require greater computational cost and tuning effort. Simpler models such as Decision Tree, k-Nearest Neighbors, Gaussian Naive Bayes, or the dummy baseline show weaker performance, underscoring the value of ensemble and linear approaches in this context.

The advantage of Random Forest over Logistic Regression in terms of Recall performance, although seemingly subtle in the cross-validated results shown in Table 1, becomes more pronounced when examined on single validation splits (as detailed in Table 2 and Table 3). This difference reflects fundamental algorithmic characteristics: Random Forest’s ensemble of decision trees can model complex, non-linear decision boundaries that adapt to local data structure, enabling better identification of minority-class compounds (BBB−) and substantially reducing false negatives. In contrast, Logistic Regression relies on a single global linear boundary, making it more susceptible to missing minority-class cases in imbalanced datasets. On single-split evaluations, Random Forest achieves significantly higher recall (0.989 with only 5 false negatives) compared to Logistic Regression (0.938 with 28 false negatives), while both models maintain comparable false positive rates (around 10%). This demonstrates Random Forest’s superior ability to handle class imbalance and improve sensitivity without sacrificing specificity, a critical advantage in drug discovery applications where missing potentially permeable compounds can be costly. Based on this comparison, Logistic Regression and Random Forest are selected for further evaluation under resampling strategies to mitigate class imbalance and enhance predictive reliability.

### 2.2. Impact of Resampling Techniques on Model Performance

The inherent class imbalance in the BBBP dataset presents a fundamental challenge for machine learning classifiers, as models tend to be biased toward the majority class, potentially compromising their ability to accurately identify blood–brain barrier permeable compounds. High recall values across most models indicate strong sensitivity to the positive class, but this often comes at the expense of precision, resulting in an increased rate of false positives. This trade-off is especially pronounced in ensemble methods such as AdaBoost and neural network architectures like MLP, which achieve strong recall but comparatively lower precision [52,53]. In drug discovery, where the costs of false positives and false negatives differ, this imbalance in predictive behavior must be carefully managed.

To address class imbalance, multiple resampling techniques are applied to the best two representative classifiers (Logistic Regression and Random Forest). These models allow evaluation of resampling effects across different algorithmic families, which include standard SMOTE, Borderline SMOTE, and an undersampling method, each designed to re-balance training data in distinct ways.

SMOTE generates synthetic minority class instances by interpolating between existing samples and their nearest neighbors, expanding the decision space of the minority class. Borderline SMOTE focuses on minority samples near the decision boundary, where misclassification risk is highest. The undersampling method reduces majority class instances while augmenting the minority class, producing balance through simultaneous contraction and expansion.

As shown in Table 2, Logistic Regression combined with SMOTE produces the strongest improvements, raising ROC AUC from 0.764 to 0.791 (+2.7%) and average precision from 0.873 to 0.887, while also improving true negative identification (82 to 93). This gain in precision is balanced by a slight drop in recall (0.938 to 0.913). Borderline SMOTE yields smaller improvements, while the combined method pushes precision slightly higher but reduces recall more substantially, showing a shift toward conservative predictions.

As documented in Table 3, Random Forest shows a stronger baseline performance and smaller changes under resampling. SMOTE slightly improves ROC AUC (+0.010) and precision, with minimal recall loss. The combined method produces the best precision (0.911) and the largest ROC AUC gain (+0.020) but lowers recall from 0.989 to 0.955, indicating a trade-off between sensitivity and specificity. Borderline SMOTE produces negligible changes.

Overall, both resampling techniques have shown improvement in terms of the prediction performance, with ROC and AUC consistently increasing across methods. Logistic Regression is more sensitive to resampling, showing larger precision–recall trade-offs, whereas Random Forest maintains stability with incremental gains. These results suggest that resampling is most useful for improving specificity and reducing false positives, which is particularly valuable in drug discovery pipelines where filtering out non-permeable compounds can save development costs and time. Subsequent analyses are based on the output from Random Forest combined with the undersampling technique.

### 2.3. Feature Ranking and Interpretation of Key Molecular Features

**Figure 1:**
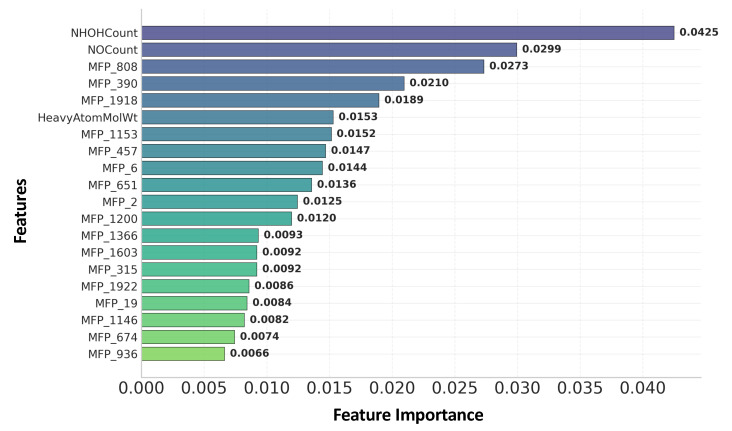
Top-ranked feature importance based on the Random Forest model.

To enhance the interpretability of the machine learning models, feature importance scores are extracted from the Random Forest classifier. This analysis highlights a combination of physicochemical descriptors and Morgan fingerprint bits as the most influential factors for BBB permeability, as shown in Figure 1.

Among the top descriptors, NHOHCount is observed to serve as the most important indicator. This feature measures the total number of hydroxyl (–OH) or amine (–NH) groups in a molecule. Each group acts as a hydrogen bond donor, increasing polarity and strengthening interactions with water or polar residues in the BBB endothelium [54,55]. However, these same interactions reduce the molecule’s lipophilicity and hinder passive diffusion through the lipid-rich membrane. Molecules with fewer donors are more likely to partition into and cross the BBB.

**Figure 2:**
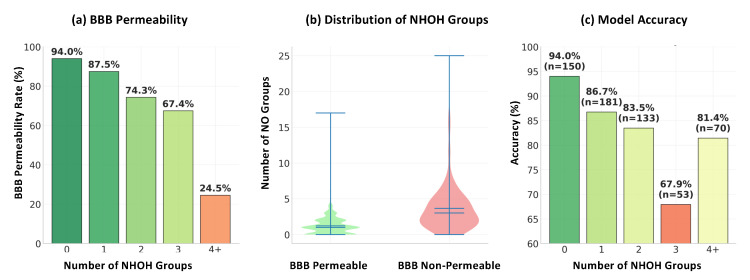
Impact of –NH/OH group count on BBB permeability: (**a**) permeability rate, (**b**) distribution across permeable and non-permeable compounds, (**c**) model accuracy.

**Figure 3:**
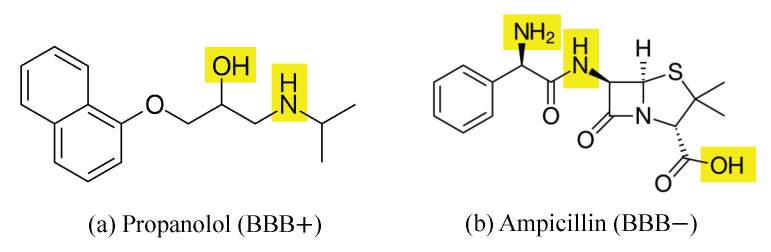
Skeletal formula of (**a**) propranolol (BBB permeable) and (**b**) ampicillin (BBB non-permeable) with –OH groups and –NH groups highlighted in yellow.

The influence of –NH/OH groups is illustrated in Figure 2. More specifically, as shown in Figure 2a, molecules with 0–1 donors achieve permeability rates above 80%, while those with four or more drop below 20%. This sharp decrease highlights the negative effect of excessive hydrogen bonding on passive diffusion. Figure 2b further illustrates that permeable compounds concentrate at low NHOH counts (median ≈ 1), while non-permeable molecules exhibit a broader spread extending into higher counts. Figure 2c quantifies the prediction complexity introduced by donor content, where accuracy systematically decreases from 94.0% (0 donors) to 67.9% (3 donors), indicating that the permeability decision boundary becomes increasingly complex as NH/OH count approaches three. However, prediction accuracy recovers for compounds with 4+ donors, as these molecules exhibit predominantly non-permeable behavior, simplifying the classification task despite their chemical complexity. Clinically, this trend is consistent with some well-known drugs such as propranolol and ampicillin. More specifically, propranolol has 2 donors and it is BBB permeable [56,57], whereas ampicillin possesses 4 donors and is poorly permeable [58,59], as shown in Figure 3, respectively. These examples confirm that the presence of a hydrogen bond donor is a critical factor of BBB permeability and must be strategically optimized in CNS drug design.

The second most important feature is NOCount, which quantifies the total number of nitrogen and oxygen atoms in a molecule. These atoms often function as hydrogen bond acceptors and increase polarity. While necessary for solubility and target binding, an excess of heteroatoms typically reduces membrane permeability [60,61]. This finding is consistent with Lipinski’s Rule of Five [62], which claims that compounds with more than 10 hydrogen bond acceptors (N and O atoms combined) rarely achieve good oral bioavailability or CNS penetration.

**Figure 4:**
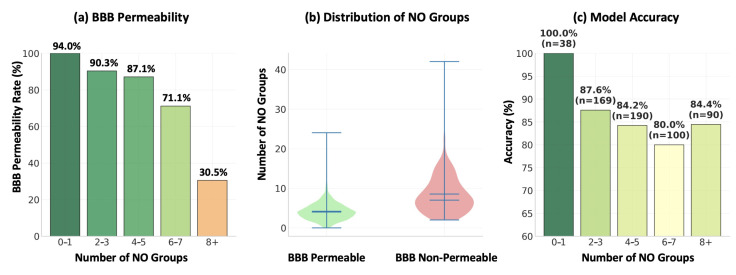
Impact of NO atom count on BBB permeability: (**a**) permeability rate, (**b**) distribution across permeable and non-permeable compounds, (**c**) model accuracy.

**Figure 5:**
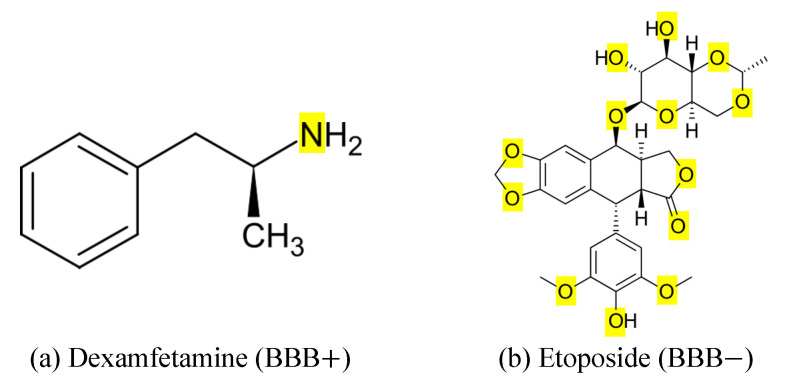
Skeletal formula of (**a**) dexamfetamine (BBB permeable) and (**b**) etoposide (BBB non-permeable) with O atoms and N atoms highlighted in yellow.

Figure 4a shows that molecules with 0–2 NO atoms achieve nearly 100% permeability, whereas those with 4 or more fall below 85%. It is further illustrated in Figure 4b that permeable compounds cluster at lower NO counts, while non-permeable molecules extend across a much wider range, including very high counts. Lastly, the model accuracy is highest (96%) for compounds with low NO counts and declines progressively with increasing heteroatom numbers, as shown in Figure 4c. Clinically, dexamfetamine with 1 N atom, shown in Figure 5a, crosses the BBB efficiently [63], while etoposide with 13 O atoms, illustrated in Figure 5b is unable to penetrate [64]. These contrasting cases highlight how balancing polarity and lipophilicity is essential in designing CNS-active drugs.

Aside from these dominant descriptors, additional features also contribute to BBB permeability prediction. HeavyAtomMolWt, a measure of molecular size, shows high importance, consistent with the principle that smaller compounds more easily diffuse through the BBB. Several Morgan fingerprint bits (e.g., MFP_808, MFP_390) also rank highly, suggesting that particular structural motifs or substructures recur among BBB-permeable drugs. Although hashed and not directly interpretable, these fingerprints likely correspond to chemical scaffolds that enhance penetration, such as small lipophilic rings or heterocycles.

Overall, the prominence of hydrogen bond donor count (NHOHCount) and heteroatom count (NOCount) confirms that polarity is the primary barrier to BBB penetration. By linking ML model-derived features to established pharmacological heuristics and real drug examples, these findings demonstrate that machine learning models capture biologically meaningful determinants of CNS drug delivery, rather than operating as black-box predictors.

## 3. Methods

### 3.1. Dataset Description and Molecular Representations

The BBBP dataset consists of 1955 unique molecules, each represented by its SMILES string, compound name, and corresponding logBB value. A threshold of logBB=−1 is applied to categorize molecules into BBB+ (permeable) and BBB− (impermeable), consistent with prior pharmacokinetic studies [24,26]. After preliminary permeability classification, the dataset contains 1492 BBB+ and 463 BBB− compounds, which confirms that the dataset is highly imbalanced. To mitigate class imbalance, SMOTE is applied exclusively within the training folds to avoid data leakage. Additional preprocessing includes removal of duplicate entries, standardization of molecular representations, and normalization of descriptor values. Compounds in the BBBP dataset are annotated by experimental BBB penetration data and are not restricted to CNS-active therapeutics, ensuring representative physicochemical diversity.

In addition to permeability information, molecular structures are encoded into numerical features using multiple complementary representations designed to capture both substructural and physicochemical properties. MACCS keys serve as 166-bit binary fingerprints, indicating the presence or absence of predefined structural fragments such as hydroxyl groups and aromatic rings. To capture more flexible substructural information, Morgan fingerprints are generated, encoding circular neighborhoods around each atom, as computed using the RDKit cheminformatics toolkit [65]. In addition to these fragment-based encodings, 208 physicochemical descriptors are calculated with RDKit, including properties such as molecular weight, lipophilicity (logP), topological polar surface area (TPSA), and counts of hydrogen bond donors and acceptors. Topological descriptors are also included to provide higher-order information on connectivity indices, ring systems, and molecular shape. All continuous-valued descriptors are standardized to zero mean and unit variance prior to model training to ensure comparability across features. These diverse representations provide a comprehensive feature set for evaluating machine learning models in BBB permeability prediction. Note that the descriptors are calculated using the dominant protonation state at physiological pH (≈7.4) [66]. As a result, the current models therefore do not explicitly consider multiple ionization states. Nevertheless, combining with machine learning, the dataset is capable of providing general guidance on BBB permeability given its wide range of properties examined.

### 3.2. Feature Engineering, Preprocessing, Model Training and Evaluation

**Figure 6:**
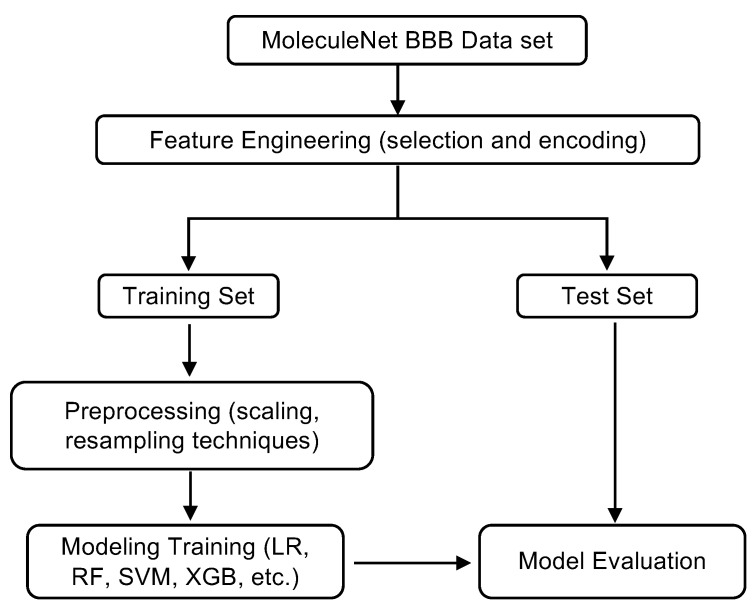
Workflow of the machine learning pipeline for BBB permeability prediction.

The overall workflow of the study is illustrated in Figure 6. Starting from the MoleculeNet BBBP dataset, raw molecular structures are transformed into numerical representations through feature engineering. This step includes encoding compounds as molecular fingerprints and physicochemical descriptors, hence capturing both substructural patterns and global molecular properties. The resulting feature matrix is then split into training and test sets, with stratification to maintain the proportion of BBB+ and BBB− classes.

To reduce redundancy and improve interpretability, feature selection is applied using feature importance scores derived from 11 different classifiers, which are further discussed in Section 2.1. Features with low variance below 0.14 threshold are excluded, thereby mitigating noise and lowering the risk of overfitting. Preprocessing of the training set involves normalization of continuous-valued features to zero mean and unit variance, as well as the application of resampling techniques to address class imbalance. In particular, oversampling methods such as SMOTE and its variants are explored to synthetically generate minority class samples, while undersampling approaches are used to further balance class representation.

Model training is conducted using a diverse range of supervised learning algorithms, each chosen to represent distinct modeling features. Logistic Regression serves as a simple yet interpretable linear baseline. Random Forest, a tree-based ensemble method, is included for its robustness to overfitting and its ability to quantify feature importance. Support Vector Machines (SVM) with both linear and radial basis function (RBF) kernels are tested for their capacity to model complex, high-dimensional decision boundaries. Gradient boosting frameworks, including XGBoost and LightGBM, are selected for their strong track record of predictive accuracy on structured data. In addition, k-Nearest Neighbors (k-NN) is evaluated as a non-parametric method relying on local molecular similarity. Model performance is evaluated through stratified ten-fold cross-validation to ensure consistent class distributions across different sampling sets.

Reliable evaluation metrics are essential in the context of BBB permeability prediction. For example, the relative consequences of false positives and false negatives differ significantly. Metrics used to evaluate the performance of the models are explained as follows. Accuracy provides an overall measure of correctness but can be misleading under class imbalance, which is the scenario of the dataset used in this study. Precision quantifies how many predicted BBB+ molecules are truly permeable, helping to minimize wasted resources on false positives. Recall or sensitivity emphasizes the correct identification of permeable molecules, reducing the risk of prematurely missing potential drug candidates. The F1-score is the harmonic mean of precision and recall, which provides a balanced assessment considering both error types.

### 3.3. Model Evaluation and Interpretation

Model performance is evaluated to capture both overall accuracy and class-specific trade-offs. In particular, accuracy, precision, recall, and F1-score are computed to assess predictive balance across BBB+ and BBB− compounds. Receiver operating characteristic (ROC) curves and the corresponding area under the curve (AUC) values quantify discriminative power, while average precision scores provide an additional summary of performance under class imbalance. To ensure robust prediction, all metrics are reported as averages across stratified ten-fold cross-validation.

To address the class imbalance present in the dataset, resampling techniques such as SMOTE, Borderline-SMOTE, and undersampling are applied prior to model training. Their impact on predictive performance is evaluated by comparing classification outcomes, including true positives, true negatives, false positives, and false negatives. This enables quantitative assessment of the trade-offs between precision and recall under different sampling strategies.

Beyond the aforementioned performance metrics, interpretability and visualization are discussed to provide chemical and biological insights into model predictions. Feature importance scores derived from the ideal ML model are used to identify the most influential descriptors. These evaluation and interpretation procedures not only benchmark model predictive power but also connect statistical performance to biologically meaningful features, offering a practical bridge between cheminformatics and drug discovery applications.

## 4. Conclusions

In this study, supervised ML models are evaluated for predicting BBB permeability using ten-fold cross-validation and resampling strategies to address class imbalance. Unlike prior work that emphasizes predictive accuracy alone, this study establishes a feature-guided interpretability framework that links model performance directly to actionable design principles for CNS drug discovery. Random Forest models achieve high F1-scores with lower computational cost than more complex gradient boosting approaches, demonstrating that algorithmic simplicity combined with transparent descriptor analysis can offer superior practical utility.

The feature analysis identifies hydrogen bond donor count (NHOHCount) and heteroatom count (NOCount) as dominant predictors, revealing clear non-linear permeability trends. It is clear that molecules with 0–1 NH/OH donors show >80% permeability, while those with four or more donors show reduced penetration. A critical decision boundary at NH/OH = 3 is identified, corresponding to a pharmacokinetic “gray zone” where predictive performance drops sharply, suggesting a structural threshold where compensatory physicochemical mechanisms become dominant. These findings provide experimentally relevant guidelines, supported by clinically established examples such as propranolol (BBB+) and ampicillin (BBB−).

From a cheminformatics perspective, NHOHCount and NOCount capture polarity, solubility, and hydrogen bonding capacity—properties that directly shape passive transcellular diffusion across the BBB. The models successfully quantify the relative importance of these features and recapitulate established pharmacological heuristics such as Lipinski’s Rule of Five, reinforcing that model predictions remain consistent with known biochemical principles rather than purely statistical correlations.

An important practical consideration is the applicability domain (AD) of these models. While stratified cross-validation indicates robust internal performance, generalization to novel chemotypes may vary. Future work could incorporate molecular similarity metrics (e.g., Tanimoto coefficient) and ensemble-based uncertainty estimates to identify predictions made outside the chemical space represented in training data. External validation on scaffold-split datasets or newly synthesized compounds will further strengthen confidence in model generalizability. Moreover, this study focuses on physicochemical descriptors associated with passive diffusion, and active transporter mechanisms such as P-glycoprotein and BCRP are not modeled. As a result, the predictions are most applicable to compounds that permeate primarily via passive mechanisms. Additionally, ionization states are not explicitly represented; incorporating pH-dependent features such as logD may further refine future predictions. Additional work includes expanding the training dataset to incorporate broader chemical diversity, exploring advanced molecular representations such as graph neural networks, and integrating predictive modeling with generative design workflows. These developments are expected to enhance both the predictive accuracy and real-world applicability of computational pipelines for BBB permeability and support more effective CNS drug discovery.

## Figures and Tables

**Table 1:** Baseline performance of selected classifiers on the imbalanced BBBP dataset.

Model	Run Time (s)	Accuracy	Precision	Recall	F1-Score
LogisticRegression	0.32	0.881	0.891	0.962	0.925
RandomForestClassifier	0.04	0.877	0.876	0.978	0.924
XGBClassifier	0.06	0.876	0.888	0.958	0.922
GradientBoostingClassifier	0.13	0.876	0.882	0.966	0.922
LGBMClassifier	0.05	0.872	0.891	0.949	0.919
MLPClassifier	0.37	0.860	0.882	0.944	0.912
AdaBoostClassifier	0.04	0.855	0.863	0.964	0.910
DecisionTreeClassifier	0.01	0.823	0.887	0.882	0.884
KNeighborsClassifier	0.00	0.814	0.844	0.928	0.884
DummyClassifier_most_frequent	0.00	0.763	0.763	1.000	0.866
GaussianNB	0.00	0.658	0.824	0.701	0.758

**Table 2:** Performance comparison of Logistic Regression with different resampling techniques. The metrics shown represent single-run evaluations and therefore differ slightly from the cross-validated results presented in Table 1.

Metric	Without SMOTE	SMOTE	Borderline SMOTE	Undersampling
True Positives (TP)	420	409 (−11)	403 (−17)	389 (−31)
True Negatives (TN)	82	93 (+11)	92 (+10)	96 (+14)
False Positives (FP)	57	46 (−11)	47 (−10)	43 (−14)
False Negatives (FN)	28	39 (+11)	45 (+17)	59 (+31)
Correct Predictions	502	502 (0)	495 (−7)	485 (−17)
Incorrect Predictions	85	85 (0)	92 (+7)	102 (+17)
Accuracy	0.855	0.855 (+0.000)	0.843 (−0.012)	0.826 (−0.029)
Precision	0.881	0.899 (+0.018)	0.896 (+0.015)	0.900 (+0.020)
Recall	0.938	0.913 (−0.025)	0.900 (−0.038)	0.868 (−0.070)
F1 Score	0.908	0.906 (−0.002)	0.898 (−0.011)	0.884 (−0.024)
ROC AUC	0.764	0.791 (+0.027)	0.781 (+0.017)	0.779 (+0.016)
Average Precision	0.873	0.887 (+0.014)	0.882 (+0.009)	0.882 (+0.009)

**Table 3:** Performance comparison of Random Forest with different resampling techniques. The metrics shown represent single-run evaluations and therefore differ slightly from the cross-validated results presented in Table 1.

Metric	Without SMOTE	SMOTE	Borderline SMOTE	Undersampling
True Positives (TP)	443	442 (−1)	440 (−3)	428 (−15)
True Negatives (TN)	87	90 (+3)	88 (+1)	97 (+10)
False Positives (FP)	52	49 (−3)	51 (−1)	42 (−10)
False Negatives (FN)	5	6 (+1)	8 (+3)	20 (+15)
Correct Predictions	530	532 (+2)	528 (−2)	525 (−5)
Incorrect Predictions	57	55 (−2)	59 (+2)	62 (+5)
Accuracy	0.903	0.906 (+0.003)	0.900 (−0.004)	0.894 (−0.009)
Precision	0.895	0.900 (+0.005)	0.896 (+0.001)	0.911 (+0.016)
Recall	0.989	0.987 (−0.002)	0.982 (−0.007)	0.955 (−0.034)
F1 Score	0.940	0.941 (+0.001)	0.937 (−0.003)	0.933 (−0.008)
ROC AUC	0.807	0.817 (+0.010)	0.808 (+0.001)	0.827 (+0.020)
Average Precision	0.893	0.898 (+0.005)	0.894 (+0.001)	0.904 (+0.011)

## Data Availability

Processed results will be available for at least 2 years after publication upon written request to the author.

## Abbreviations

The following abbreviations are used in this manuscript:

AUC	Area Under the Curve
BBB	Blood-Brain Barrier
BBBP	Blood-Brain Barrier Penetration
CNS	Central Neural System
GaussianNB	Gaussian Naive Bayes
k-NN	k-Nearest Neighbors
LightGBM	Light Gradient Boosting Machine
logP	Lipophilicity
MACCS	Molecular ACCess System
MD	Molecular Dynamics
MFP	Morgan FingerPrint
MLP	Multi-Layer Perceptron
RBF	Radial Basis Function
ROC	Receiver Operating Characteristic
SMILES	Simplified Molecular Input Line Entry System
SMOTE	Synthetic Minority Oversampling Technique
SVM	Support Vector Machines
TPSA	Topological Polar Surface Area
XGBoost	eXtreme Gradient Boosting
